# Healthcare inequities in Chinese megacities: the older adult population’s accessibility to public hospitals in suburban Shanghai

**DOI:** 10.3389/fpubh.2025.1700098

**Published:** 2025-12-18

**Authors:** Chunlan Wang, Chen Li, Anni Zhang, Gaoxiang Gu, Shangguang Yang

**Affiliations:** 1 School of Social Development, East China Normal University, Shanghai, China; 2 Department of Social Sciences and Policy Studies, The Education University of Hong Kong, Ting Kok, Hong Kong SAR, China; 3 School of Social Development and Public Policy, Fudan University, Shanghai, China; 4 School of Business, East China University of Science and Technology, Shanghai, China

**Keywords:** health equity, health inequality, hospital accessibility, older adult, Shanghai, China

## Abstract

**Introduction:**

Due to the unbalanced spatial distribution of healthcare resources, the suburbanization of the older adult population may contribute to new health inequities. This study investigates public hospital accessibility of different regions and groups in Shanghai, exploring the equitability of resource distribution within the broader context of the city’s evolving spatial organization and political economy.

**Methods:**

Drawing on data from the 2000 and 2010 population censuses and the geographical distribution of hospitals, this study employs two complementary measures: the healthcare supply and demand matching index, and the average distance to the closest hospital, evaluating both the diversity of healthcare options and geographical convenience.

**Results:**

The findings reveal significant healthcare inequities in public hospital accessibility: (1) Continuous increase in the number of healthcare hospitals has brought about some improvements in supply levels, but in suburban Shanghai, the accessibility of public hospital is rather poor, and the average distance for all older adults to the closest hospitals has shown almost no improvement, increasing from 2.3 km in 2000 to 2.4 km in 2010; (2) older adults in suburban areas face greater distances to the closest hospital compared to their counterparts not in suburban areas. In 2010, the average distance was 4.4 km for older adults outside the outer-ring, compared to only 0.9 km within the inner-ring; and (3) there is a widening gap in health inequities among individuals from different socioeconomic strata between 2000 and 2010. The distance to the closest tertiary hospital increased by approximately 6 km for older adults in blue-collar communities, but only 1 km for those in white-collar and advanced white-collar communities.

**Conclusion:**

The study concludes that the inequitable accessibility to public hospitals for suburban older adults is a profound structural issue in Shanghai. Policy interventions must extend beyond simply increasing hospital numbers to address the root causes in healthcare governance and urban spatial planning to achieve health equity.

## 1 Introduction

Access to healthcare services has been at the center of focus in health geography. It is often associated with the location of healthcare resources provision and the location of people who are in need of healthcare (1), such as those people with chronic diseases (2). Adequate access to essential healthcare resources is important to investigate health inequities between different groups of people defined geographically or socioeconomically (3) and therefore poses significant institutional challenges for governments across the globe to tackle (4). For example, in India, due to poor accessibility, scheduled caste, which is the lowest level in the hierarchy and mostly living in rural areas, has higher risk of mortality (5). In Canada, health inequities across the continuum of rural–urban exist; those in urban areas are mostly likely to receive specialist physician services, while those in rural areas have rarely received a flu shot and seen regular medical doctor (6). Such issues have become more prominent during the COVID−19 pandemic. For example, Njoku et al. (7) find that in the US, due to limited vaccination clinic availability in certain neighborhoods, racial and ethnic disparities in COVID−19 vaccine uptake are prevalent.

In China, the mismatch between the increasing healthcare demand and safe and effective healthcare supply has been one of the most pressing social problems. Such a mismatch, as Yu et al. explain, is the result of the uneven geographical distribution of healthcare services, as most large and high-quality healthcare institutions are concentrated in more developed areas (8). This has produced barriers for those people who live in less developed areas and have lower socioeconomic status to access well-established healthcare services (9, 10). For example, in the context of the ongoing process of population suburbanization (11), substantial people, especially those disadvantaged such as low-income group and older adults (12), have moved to suburban areas, where have poorer public services and facilities (13). Suburbanization has therefore affected the well-being of the residents (14), resulting in significant inequities in terms of accessibility to public resources (15, 16). In the sense, there is a growing need to examine whether healthcare services accessibility is equitable for disadvantaged people who live in suburban China. This requires a comprehensive quantitative evaluation on the equitability and a clear understanding of the concept of health equity, which remains under-investigated in China.

This research investigates the equitability of public hospitals accessibility for the older adult population in suburban Shanghai. The accessibility was measured through two ways: the healthcare supply and demand matching index and the average distance of the older adult population to the closest hospital. The research pays particular attention to the older adult population because as the world’s most populous country, China’s population aging is at an extraordinarily faster rate than many other countries (17), and there is a growing concern over the increasing healthcare demand of the older adult population in the country (18). The concept of health equity contributes to the investigation as it concerns the great differences in health that are considered unnecessary, avoidable, unjust and unfair through its moral and ethical dimension (19), and is then tied to political economic system, and institutional setting that induce inequities (20). The research therefore offers new insights to the equitability of healthcare services accessibility by articulating it with the needs and rights of disadvantaged people within the context of urban China’s healthcare governance mode and political economic system.

## 2 Health inequity and healthcare services accessibility in urban China

Substantial researchers have made increasing efforts to distinguish the two terminologies, health inequality and health inequity (19). Health inequality denotes the differences in health outcomes of both individuals and social groups based on socioeconomic status, geographical location, gender, age, race/ethnicity and other factors (21). In contrast, health inequity is an ethical and judgmental concept and focuses on systemic health disparities between individuals and social groups (22). Specifically, in an inequitable situation, the causes of health differences are out of individuals’ and social groups’ direct control but determined by broader political, economic and social contexts (23). With unfair and unjust distribution of power, an inequitable situation may put those already disadvantaged individuals and social groups in a more disadvantaged condition with respect to their health (24). In this sense, reducing or eliminating health inequities and deploying available resources to benefit the whole population is an issue of social justice and emphasizes the political economic dimension of health problems (25).

In China, there is deteriorating and inequitable access to healthcare resources (26). For example, at the national scale, Chen et al. (27) identified that the flawed government policies prohibit the flow of public healthcare resources to rural China. Pan and Shallcross (28) found that provinces in western China are disadvantaged in healthcare resources distribution due to the slower local economic development and limited public sector investment. Yan et al. (29) unveiled the increasing divergence in the distributions of physicians and healthcare beds. Cities with a high physician-to-bed ratio are often located in developed areas and have high administrative level, strong financial capacity, advanced medical technologies and few primary care facilities, while less developed areas are unable to attract physicians. Jia et al. found that approximately 30% of the total population in China had no access to Primary Healthcare Centres (PHCs) within their 6 km catchment areas and approximately 68.4% of the total population had no access to PHCs within their 1.5 km catchment areas (30). At the individual or social group scale, Chen et al. (31) demonstrated that high-income individuals generally benefit the most from government healthcare subsidies. Tao et al. (32) revealed that compared with the car-mode subgroup, the transit-mode subgroup is disadvantaged in healthcare services accessibility in Shenzhen.

In the context of the population aging in China, scholars have started to focus on the older adults’ accessibility to healthcare resources. One body of research aims at measuring health inequalities among and between different provinces and cities in China. For example, Yang et al. (33) found that the spatial distribution of health inequalities for older people corresponds to the spatial distribution of economic development in China. Jia et al. (30) found that more developed municipalities/provinces like Shanghai, Beijing, Jiangsu and Zhejiang had higher proximity to the nearest PHCs, while less developed provinces like Tibet, Guizhou and Guangxi had lower proximity. Yan et al. (34) found that in Hefei, older adults were disadvantaged in traveling long distances to access cross-city healthcare resources due to the uneven healthcare resources distribution. Another body of research pays attention to the influencing factors of healthcare services utilization. Evandrou et al. (35) found that those older adults, who are women and rural residents, lack sufficient education, have no individual income sources, and are ex-smokers and from poor economic status households, are more likely to report disability and have poor self-rated health. Gong et al. (36) found that those older people who are well-educated, communist party members, urban residence and have better financial situations are more likely to have physical examinations or inpatient care. Also, Wang et al. (37) and Fu et al. (38) found a strong pro-rich inequality in healthcare services utilization among the older adult population.

In China, it has been widely acknowledged that the equity of healthcare resources accessibility is an issue related to the complex health system. First, governance structure in the health system is highly fragmented (39). Not only is the National Health Commission responsible for people’s health, but various other departments, such as the Ministry of Finance and National Development and Reform Commission, also have the authority to govern healthcare resources. This has led to competing interests and conflicting policies over health issues between different departments and unclear development objectives of public healthcare institutions (8). Second, in the post-reform period, with the decentralization process that has transferred local resource mobilization and service provision powers to local governments, healthcare resources supply has become profit-driven (40). For example, a large proportion of physicians’ income in public hospitals is from their profitable performance based on the revenue they generated (41, 42). This has incentivized hospitals and physicians to pursue profits as their top priorities by over-prescribing drugs and over-using high-tech diagnostic tests (43). Furthermore, despite the market reform, public hospitals are still governed by the bureaucratic system. Hospital directors and other managerial personnel are appointed by relevant government departments and hence only accountable to the government departments; and government funding plays a significant role in operating public hospitals.

While existing studies have explored issues of health inequality in resource distribution, they often fail to distinguish the connotations between health inequality and health inequity. This oversight has hindered a deeper understanding of the structural determinants embedded in the political economy of Chinese megacities, which transform apparent differences into systemic injustices. In the empirical section, the study first quantifies the health inequalities in public hospital accessibility for older adults in suburban Shanghai. Using neighborhood committee (*juweihui* in Chinese; the lowest semi-administrative unit in urban China) as the basic research unit and comparing the accessibility in 2000 and 2010 through quantitative analysis, the study investigates the matching between the increasing healthcare demand of the older adult population and the spatial distribution of public hospitals over time. Based on these empirical findings, the study critically interrogates the health inequities inherent in this spatial mismatch. Our analysis ultimately demonstrates that these observed disparities are not neutral outcomes but are rendered inevitable by the unique healthcare governance mode and political economic system in urban China. Therefore, we frame these inequalities as manifestations of structural inequity. These findings deepen both the theoretical and practical understanding of healthcare inequities in the context of suburbanization and offer a inspiration for reforming healthcare policy and urban governance.

## 3 Materials and methods

### 3.1 The case city

Shanghai is selected as the case city. Among Chinese mega cities, Shanghai has the most aging population. In 2020, in Shanghai, 25.6% of the total population were older adult population (aged over 65 years old)^1^ (44). This number almost doubled the national data (13.5%) (45). Shanghai is also facing insufficient high-quality healthcare services supply. For example, the municipal government of Shanghai in its Regional Health Planning of Shanghai Municipality (2011–2020) pointed out that the issues related to the accessibility to healthcare services are still serious (46). Shanghai therefore is a great case to study the equitability of public hospitals accessibility for the older adult population.

**Figure 1:**
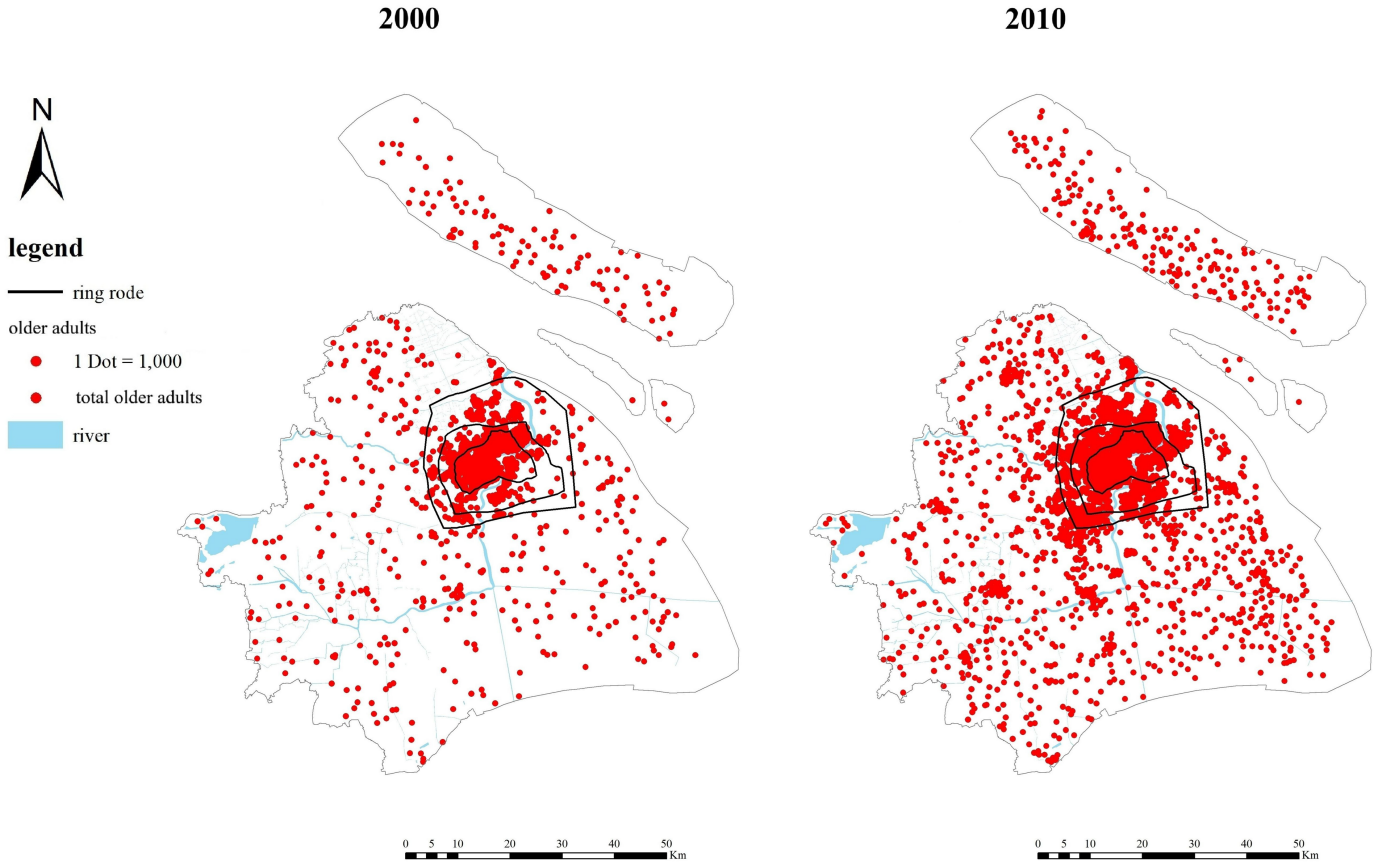
The distribution of the older adult population in Shanghai in 2000 and 2010.

Shanghai can be divided into three zones: inner-ring, mid-ring and outer-ring. Outside the outer-ring is Shanghai’s suburban areas occupying 90% of Shanghai’s administrative region. Within the outer-ring is Shanghai’s built-up areas including the urban fringes (between inner-ring and outer-ring) and the central areas (within the inner-ring). As Figure 1 shows, in the urban fringes, from 2000 to 2010, the proportion of total population decreased slightly, while the proportion of the older adult population increased by 6%. In 2000, 40.2% of the total population was distributed in the suburban areas. The number increased to 50.4% in 2010. In 2000, 37.4% of the total older adult population was distributed in the suburbs. In 2010, the number increased to 41.6%. In 2010, the proportions of both the total population and the older adult population within the inner-ring showed a declining trend. This shows a significant suburbanization of the older adult population in Shanghai.

**Figure 2:**
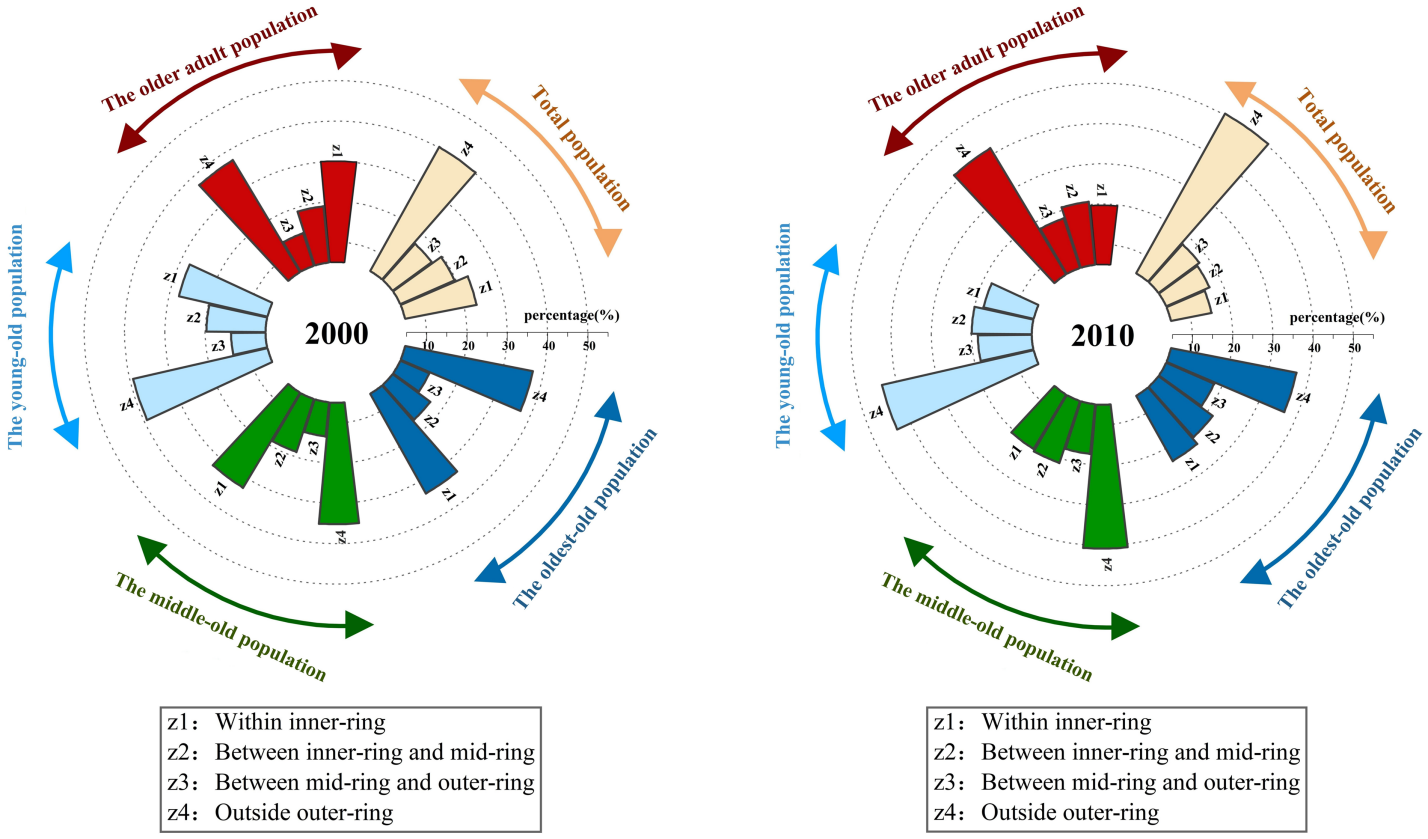
The change of the distribution of the older adult population in different zones in Shanghai.

**Figure 3:**
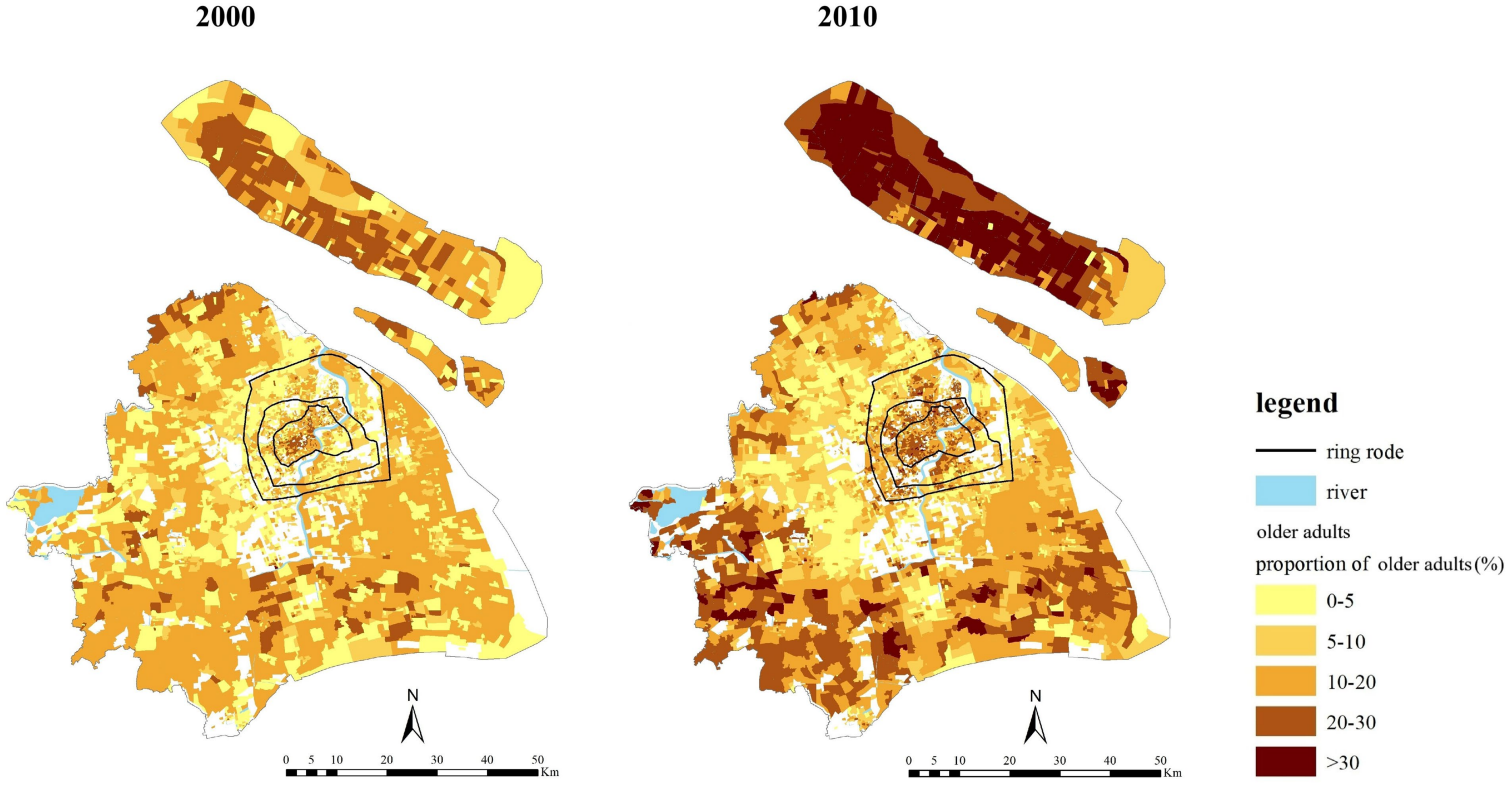
The spatial differences of the aging degree of communities in Shanghai in 2000 and 2010.

As Figure 2 shows, there were differences in the distribution of suburbanization among the older adult population in different age groups^2^. From 2000 to 2010, the proportion of the oldest-old population within the outer-ring increased slightly, while the proportion of the young-old and middle-old population in the area decreased. In 2010, the proportion of the older adult population distributed in the suburbs from high to low was the young-old population, the middle-old population, and the oldest-old population, respectively. As Figure 3 shows, from 2000 to 2010, ‘deep aging’ communities (more than 30% of the population is the older adult population) increased rapidly in both the central and the suburban areas. Compared with 2000, in 2010, the aging degree of the communities in the suburban areas deepened; and the communities were mostly far away from the central areas and on the outer edges of Shanghai’s administrative region, such as in Chongming district, Jinshan district, Qingpu district, Pudong New District. This again shows a significant trend of the older adult population suburbanization in Shanghai.

### 3.2 The data sources

The data sources include China’s population census in 2000 and 2010, Shanghai Municipal Human Resources and Social Security Bureau, Shanghai Urban Geographical Information Database, and Shanghai hospitals dot layer. From these sources, we obtained the population data, the hospital list and distribution, and the information on the distance between hospitals and communities. Until May 2019, there were 1,637 designated healthcare institutions with medical insurance in Shanghai. Considering the actual services provided by different categories of healthcare institutions to the older adult population, maternal and child, pediatric, mental health after deleting community healthcare services centers, healthcare clinics, rehabilitation centers, nursing homes, rehabilitation hospitals, medical examination centers affiliated to hospitals, and healthcare institutions whose year of incorporating into medical insurance cannot be found, were excluded from the analysis. Therefore, the healthcare institutions included in the analysis were 184 hospitals in 2000 (55 tertiary hospitals and 129 secondary hospitals), 218 hospitals in 2010 (63 tertiary hospitals and 155 secondary hospitals), and 276 hospitals in 2019 (80 tertiary hospitals and 196 secondary hospitals). These hospitals are all incorporated into Shanghai’s medical insurance system and categorized into secondary and tertiary hospitals according to the medical expense reimbursement. Distance between the geometric center of the communities where the older adult population resides and the hospitals were adopted to measure the accessibility. Due to the lack of traffic layer, the distance of the traffic network was not considered in the analysis. Given that Shanghai has flat terrain and a relatively developed traffic network, it is unlikely that the distance will have a great impact on the results.

**Figure 4:**
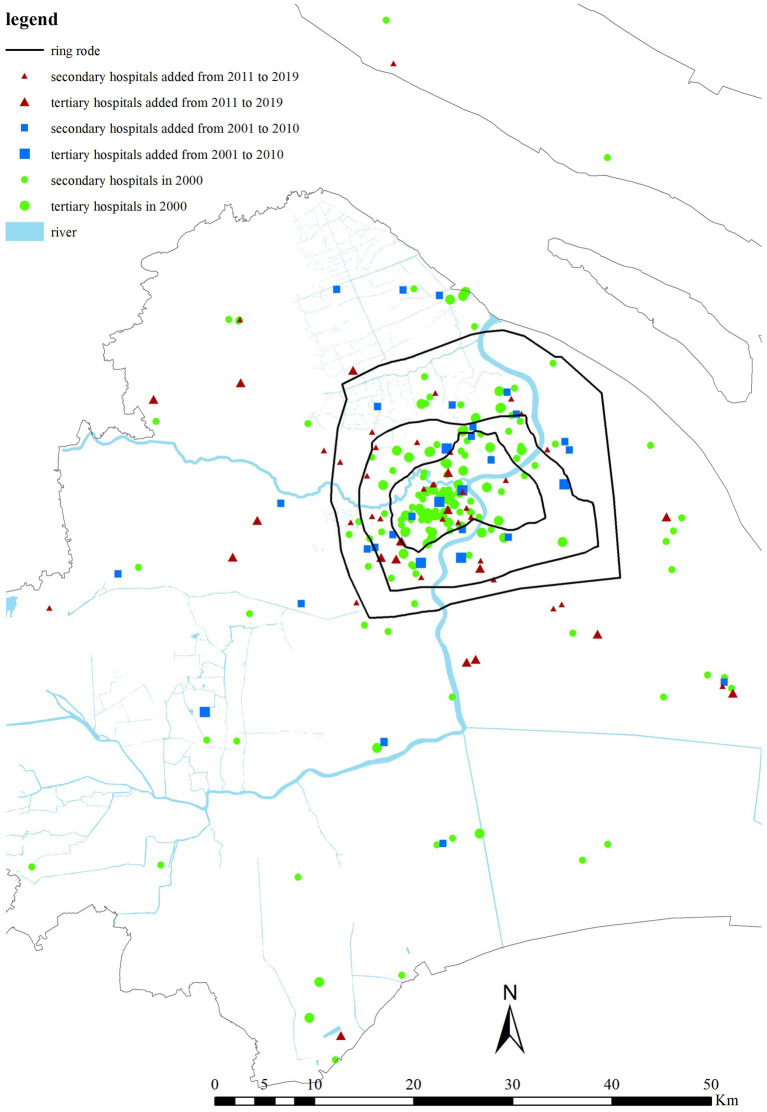
Distribution of hospitals at different levels in Shanghai from 2000 to 2019.

**Figure 5:**
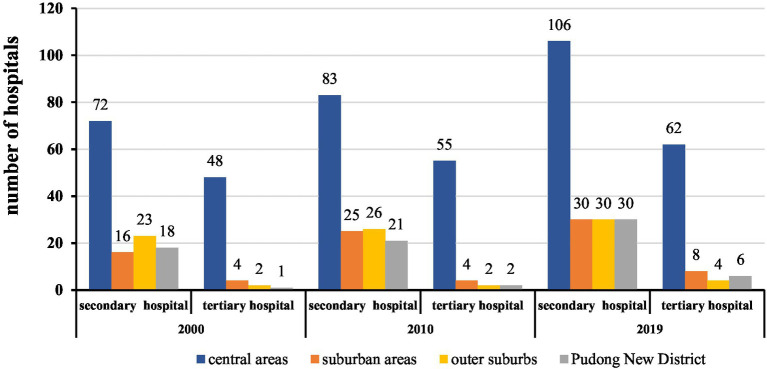
Distribution of secondary and tertiary hospitals in Shanghai in 2000, 2010, and 2019.

As^3^ Figures 4, 5 shows, while the distribution of healthcare institutions has been spreading to the suburban areas since 2000, compared with 2000, in 2010, there were still more tertiary hospitals within the inner-ring. By 2019, the numbers of secondary and tertiary hospitals in the city center and suburbs increased simultaneously, with relatively little increase in the suburbs. In general, the distribution pattern of hospitals in the city center and suburbs in Shanghai has not changed fundamentally, and the suburbs have not yet become the key area of building new hospitals.

**Figure 6:**
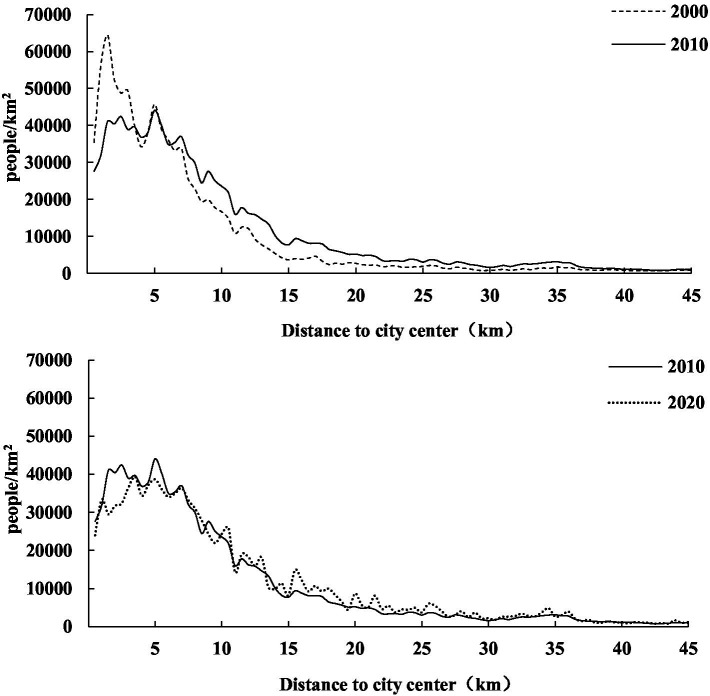
Population density by distance from Shanghai city center in 2000, 2010, and 2020.

It is noted that although the seventh population census data have been released, the demographic data at *juweihui* scale by age structure is not yet available. This is largely due to the fact that the central government has recently strengthened the control of the geocoded spatial and temporal data and no longer open such microscopic scale population data for academic uses. Despite the unavailability of detailed 2020 census data at the *juweihui* scale, the macro-scale spatial pattern shown in Figure 6 provides compelling evidence for the continuation of the suburbanization trend. The 2020 population density curve largely retains the shape of the 2010 curve and 2000 curve, indicating a stable overall distribution. Furthermore, the 2020 distribution shows a discernible outward shift, with both the inner and outer suburbs experiencing a slight increase in population density compared to 2010. Meanwhile, according to the latest data of healthcare institutions collected in 2019 (see Figures 4, 5), the distribution of public hospitals did not undergo a fundamental reorientation toward the suburbs. This persistent spatial mismatch between a suburbanizing population and a centrally concentrated hospital system suggests that the healthcare accessibility inequities we identified for 2010 have not been alleviated but likely intensified. Therefore, the unavailability of the 2020 population census data would not weaken our central conclusion regarding intrinsic structural inequities.

### 3.3 The healthcare service’s accessibility index

This study employs two complementary measures to evaluate healthcare accessibility, one is the supply and demand matching index, the other one is the average distance to the closest hospital. Each measure is designed to capture a distinct dimension of accessibility, the former emphasizes diversity of choice under ideal conditions, while the latter focuses on convenience in practical scenarios. Their combined use enhances the robustness of the evaluation.

The supply and demand matching index is based on the assumption that, ideally, residents living anywhere in Shanghai can access healthcare services provided by any hospitals in the city, regardless of distance or other constraints. Accordingly, this index is constructed based on a normative assumption of free choice and aims to assess the diversity of hospital options available to residents. All hospitals included in the analysis are general secondary or tertiary hospitals, which offer more comprehensive healthcare services than clinics or community health centers and are capable of meeting the basic healthcare needs of the population. Although this assumption may not fully reflect real-world constraints such as travel costs or individual preferences, it provides a useful benchmark for assessing spatial equity in an idealized context.

In contrast, the average distance to the closest hospital adopts a more practical assumption that residents tend to choose the closest secondary or tertiary hospital for medical treatment, emphasizing the importance of geographical convenience. This measure reflects the common behavior, particularly among older adults, of prioritizing proximity when choosing healthcare services, thereby directly capturing spatial barriers shaped by travel costs and mobility constraints. By integrating both measures, this study reduces the risk of over-reliance on a single assumption and offers a more nuanced, multifaceted perspective on healthcare accessibility.

Based on the first assumption, we calculated the cumulative service supply intensity of all hospitals in Shanghai to each *juweihui* as the healthcare services supply index^4^, and classified it into five levels (assigned 1–5 points according to the service supply intensity from weak to strong), which is represented by Equation 1:


(1) $$\;S_{\mathrm{xy}}=\frac{20\%\sum_{i=1}^{n}d_{2i}+80\%\sum_{j=1}^{m}d_{3j}}{20\%+80\%}$$

*S_xy_* is the healthcare services supply intensity of the *x^th^ juweihui* in *y^th^* year, where *y* = 2000 or 2010. *d_2i_* is the distance from the *juweihui* to the *i^th^* secondary hospital, where *i* = 1, 2, 3, … to *n*, *n* is the total number of secondary hospitals. *d_3j_* is the distance from the *juweihui* to the *j^th^* tertiary hospital, where *j* = 1, 2, 3, …to *m*, *m* is the total number of tertiary hospitals. Twenty is the weight of secondary hospitals; 80% is the weight of tertiary hospitals^5^.

These two weights, 20% for secondary hospital and 80% for tertiary hospital, were assigned according to the ratio of medical staff to hospital beds, as published on the websites of representative secondary and tertiary hospitals in Shanghai. A comparison of these ratios revealed that the tertiary hospitals generally have about 4 times as many medical staff and beds as secondary hospitals. In some well-known tertiary hospitals, this ratio is even higher, ranging from 8 to 10 times more. For example, Dahua Hospital in Xuhui District is a secondary hospital with 360 beds and 355 medical staff, while Minhang Central Hospital in Minhang District is a tertiary institution that operates on a significantly larger scale with more than 900 beds and over 1,500 medical staff. Furthermore, Shanghai Central Hospital, a major teaching hospital of the Ministry of Health, surpasses even this level with more than 1,700 beds and 3,217 medical staff. Given the limited number of the well-known tertiary hospitals in Shanghai, we decided to assign that the weight of tertiary hospitals is 4 times more than that of secondary hospitals. This weighting is further supported by the actual number of patients. The national outpatient statistics in 2023 indicate that tertiary hospitals account for 82% of patient visits, while secondary hospitals account for 17% (47), a distribution that aligns closely with our predefined weights. This consistency between resource allocation and actual patient choice provides dual validation of the weighting scheme, reflecting both the structural capacity of different hospitals and the revealed preferences of the population.

The healthcare services demand index is mainly composed of a weighted average of four indicators^6^, including degree of aging (the older adult population/permanent population), degree of old aging (the oldest-old population/the older adult population), unhealthy proportion (the number of unhealthy older adult population/total count of health status), and occupation index^7^ (Table 1). The scores of the four indicators are multiplied by their respective weights and added up to obtain the comprehensive medical demand, which is expressed by Equation 2:


(2) $$\;D_{\mathrm{xy}}=\frac{\sum_{i=1}^{n}s_{i}w_{i}}{w_{1}+w_{2}+\ldots +w_{n}}$$

**Table 1:** The composition of healthcare services demand index.

Dimension	Weight	Grade	Point
Degree of aging (the older adult population/permanent population)	45%/30%	<5%	1
		5–10%	2
		10–20%	3
		20–30%	4
		≥30%	5
Degree of old aging (the oldest-old population/the older adult population)	45%/30%	<10%	1
		10–15%	2
		15–20%	3
		20–25%	4
		≥25%	5
Unhealthy proportion (the number of unhealthy older adult population/total count of health status)	30%	<5%	1
		5–10%	2
		10–15%	3
		15–20%	4
		≥20%	5
Occupation index	10%	>4.8	1
		3.5–4.8	3
		≤3.5	5

*D_xy_* is the healthcare services demand intensity of the *x^th^ juweihui* in *y^th^* year, where *y* = 2000 or 2010. *s_i_* is the score of the *i^th^* dimension (Table 1). *w_i_* is the weight of the *i^th^* dimension. *n* is the total number of dimensions in a certain year.

The supply and demand matching index is equal to the demand index minus the supply index for each juweihui and ranges from -5 to 5, as shown in Equation 3:


(3) $$M_{\mathrm{xy}}=D_{\mathrm{xy}}-S_{\mathrm{xy}}$$

*M_xy_* is the supply and demand matching index of healthcare services in different *juweihui* and different years. According to the respective interval of the supply and the demand and the matching situation, we divided the communities into nine types. The degree of matching from high to low is as follows^8^ (see Table 2).

**Table 2:** Matching types of healthcare services supply and demand.

Community matching categories	Types	*M*_xy_	*D*_xy_	*S*_xy_
high matching degree of the supply and the demand	High demand and high supply	−1 ~ 1	4, 5	4, 5
	Medium demand and medium supply	0	3	3
	Low demand and low supply	−1 ~ 1	1, 2	1, 2
Medium matching degree of the supply and the demand	High demand and medium supply	1 ~ 2	4, 5	3
	Medium demand and low supply	1 ~ 2	3	1, 2
	Medium demand and high supply	-2 ~ −1	3	4, 5
	Low demand and medium supply	-2 ~ −1	1, 2	3
Low matching degree of the supply and the demand	High demand and low supply	2 ~ 4	4, 5	1, 2
	Low demand and high supply	−4 ~ −2	1, 2	4, 5

Also, we used the distance of the older adult population to the closest medical institution to evaluate the accessibility to the healthcare services and the differences between the accessibilities of the older adult population from different socioeconomic strata and the dynamic change characteristics. We used the distance-based method to calculate the accessibility of the older adult population to medical institutions^9^. The smaller the distance, the higher accessibility of the older adult population to medical institutions. At the same time, the weighted standard deviation was calculated to show the difference of medical distance within the group, as shown in Equations 4, 5.


(4) $$\mathrm{Di}s_{m}=\frac{\;\sum_{i=1}^{n}p_{c,i}*d_{i}\;}{p_{t}}$$

(5) $$\;\mathrm{Di}s_{\mathrm{st}}=\sqrt{\frac{\;\sum_{i=1}^{n}p_{c,i}*{\left(d_{i}-\mathrm{Di}s_{m}\right)}^{2}\;}{p_{t}}}$$

*Dis_m_* is the weighted average distance of the older adult population to the closest medical institutions. *Dis_st_* is the weighted standard deviation distance of the older adult population to the closest medical institutions. *p_c,i_* is the total number of older adults of a certain group in community *i*. $p_{t}$ is the total number of older adults in a certain group in the city. *d_i_* is the distance from community *i* to the closest hospital.

## 4 Results

### 4.1 The distribution of healthcare services supply and demand index

**Figure 7:**
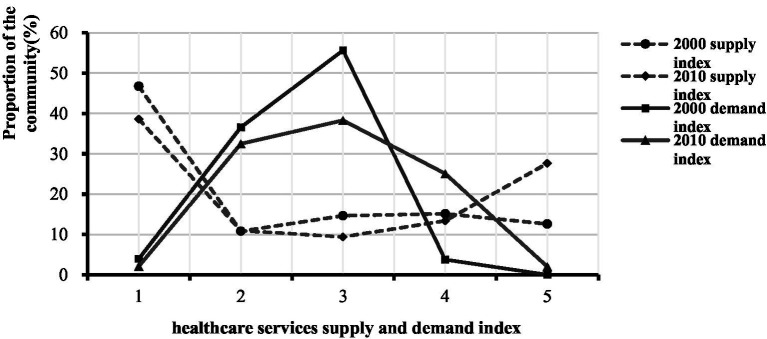
Distribution characteristics of the healthcare services supply and demand index.

**Figure 8:**
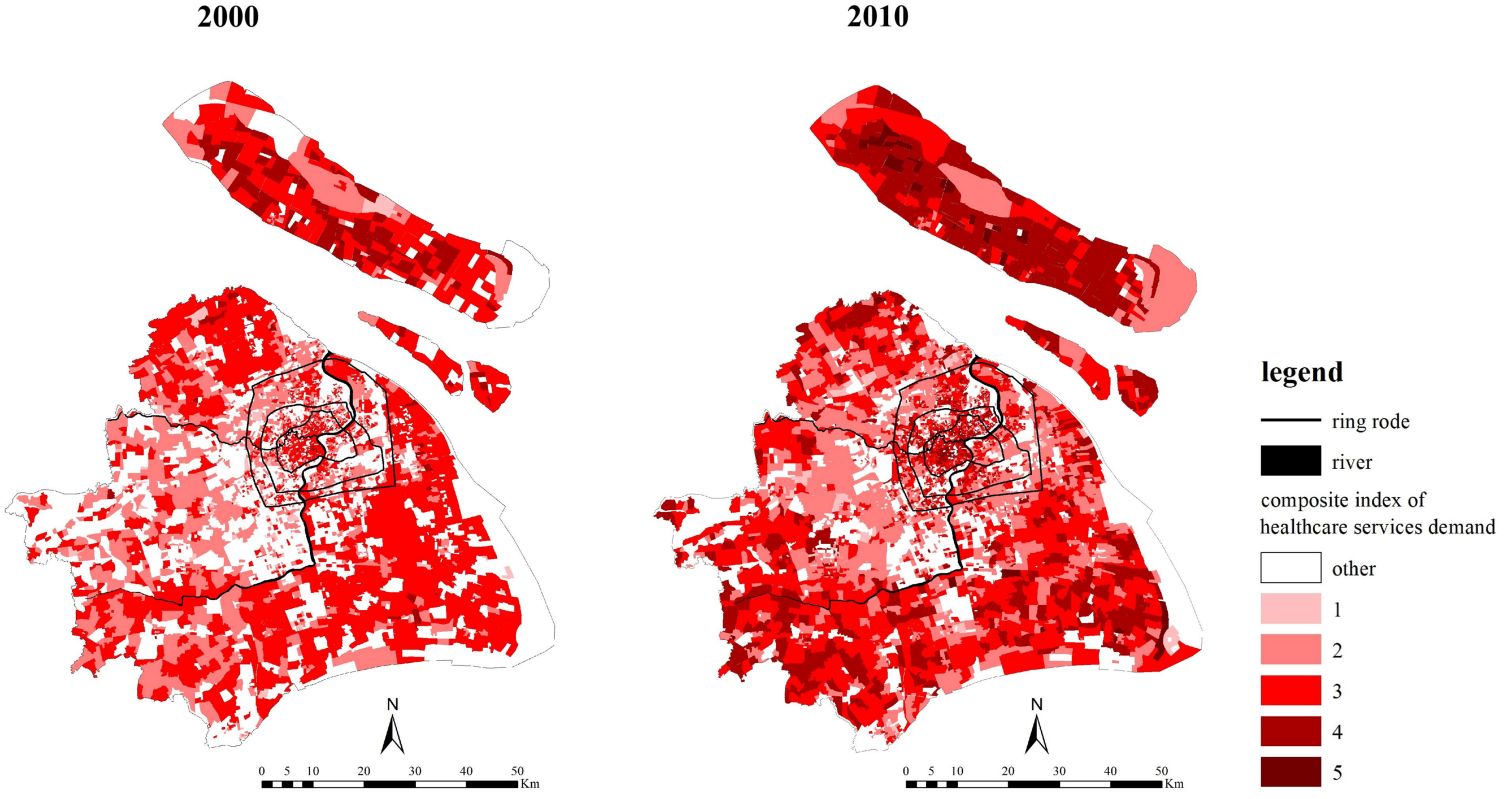
The interpolation of the older adult population healthcare services demand in 2000 and 2010.

The healthcare services demand index of the older adult population in each community had roughly normal distribution characteristics (Figure 7). In 2010, the distribution curve of the demand index changed rapidly. Low demand communities with an index of less than or equal to 3 decreased, while high demand communities with an index of more than 4 increased rapidly. The distribution pattern of the supply intensity is quite different from that of the demand index. The number and proportion of communities with low supply intensity was relatively large, while the number of communities with high supply intensity was small. In 2010, the proportion of the number of communities with middle and low supply index decreased, while the proportion of the number of communities with high level of supply index increased. This indicates that the situation of some communities with the lowest supply level had been improved, and at the same time, the situation of communities with moderate supply intensity had been further enhanced. Communities with high demand were relatively concentrated within the inner-ring and outside the outer-ring (Figure 8). The communities with high demand expanded both within and outside the outer-ring; the expansion to outside the outer-ring was especially fast.

**Figure 9:**
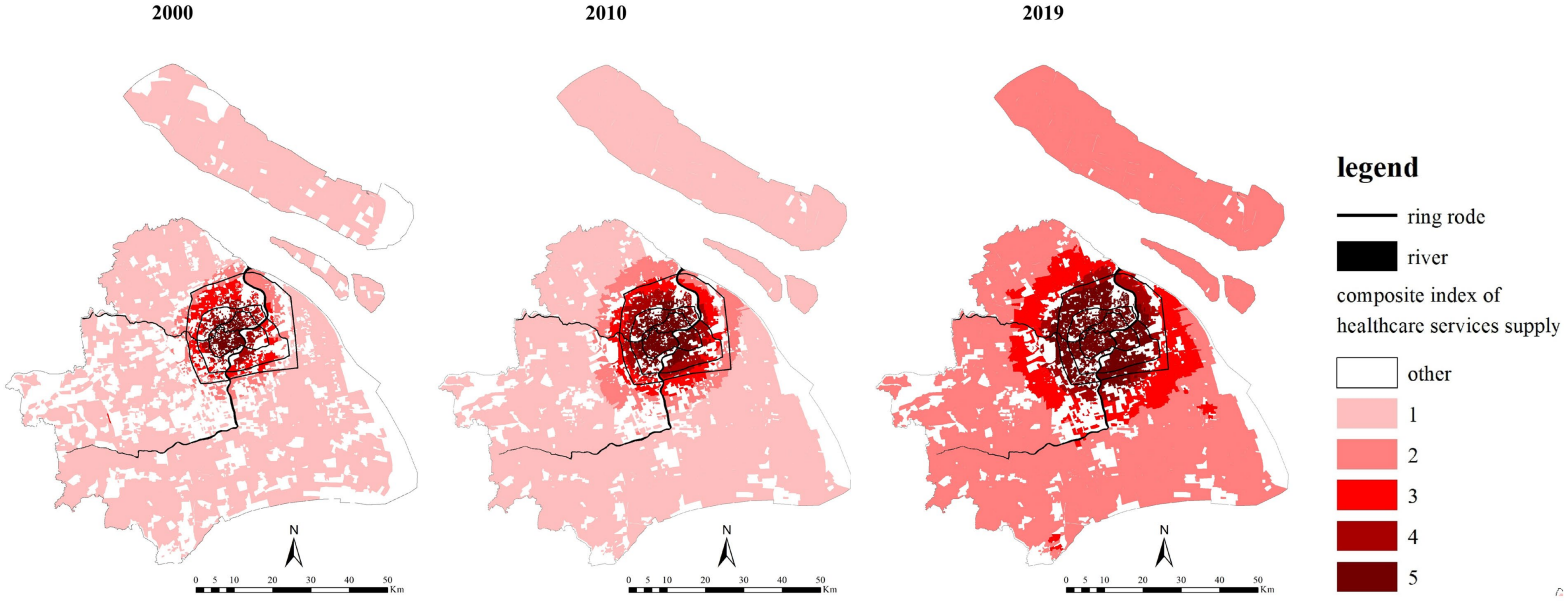
The interpolation of the older adult population healthcare service supply in 2000, 2010 and 2019.

Figure 9 shows that in the past 20 years, there were obvious differences in the level of healthcare services between different areas in Shanghai, and the central areas had the highest level of healthcare services. The distribution of communities with high supply intensity and high-level of healthcare services expanded to the suburbs in the past years. By 2019, communities within the outer-ring were the communities with high service supply levels. The changes in the supply level between the inner-ring and outer-ring exactly corresponded to the transfer process from the medium-level range to the high-level range. In sharp contrast to the population suburbanization and the multi-center urban structure, the supply intensity shows a strong single-center circle structure.

### 4.2 The distribution of healthcare services supply and demand matching index

**Figure 10:**
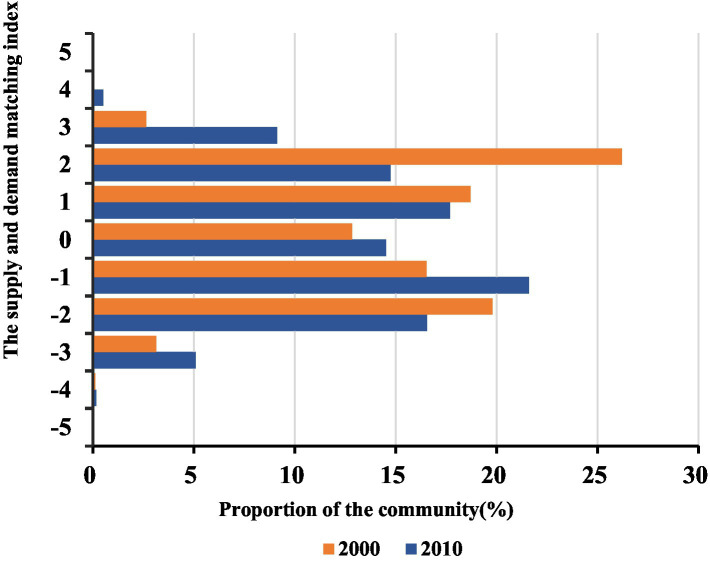
The supply and demand matching index of healthcare service in 2000 and 2010.

As Figure 10 shows, there are relatively few communities in Shanghai that have large differences between the demand and supply. Specifically, the proportion of communities with the supply and demand matching index greater than 3 or less than −3 was less than 1%, and about half communities had the matching index between −1 and 1.

**Figure 11:**
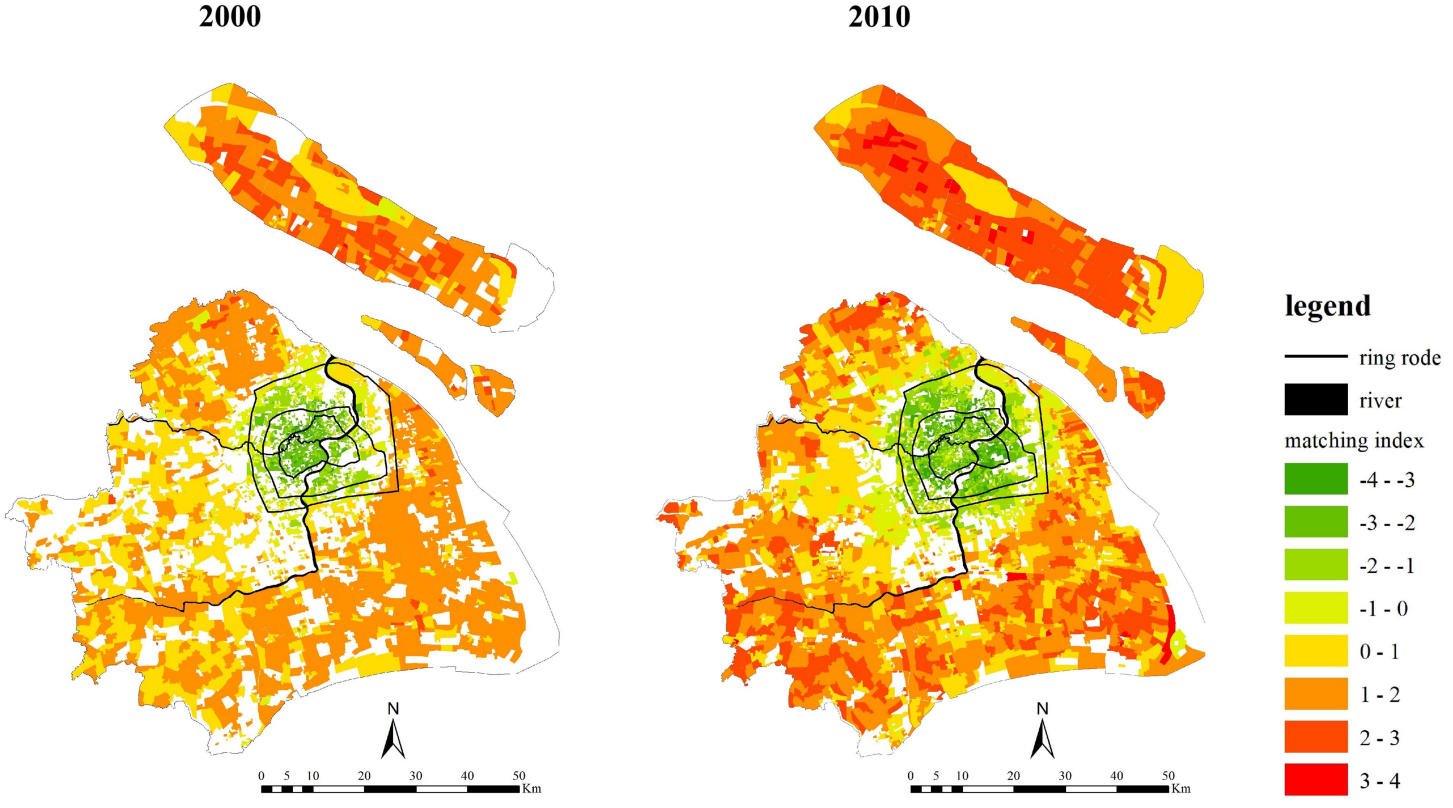
The interpolation of the older adult population healthcare service supply and demand matching index in 2000 and 2010.

From 2000 to 2010, the proportion of communities where the demand is greater than the supply decreased, while the proportion of communities where the supply is greater than the demand increased. The ranges of the decrease and the increase was small, within 5%. As Figure 11 shows, mainly located in the central areas, these communities’ supply had long been sufficient. The proportion of communities that have the matching index greater than 3 increased rapidly due to the aging suburban areas and its relatively poor healthcare services. In other words, the supply cannot meet the demand in the suburban areas.

**Figure 12:**
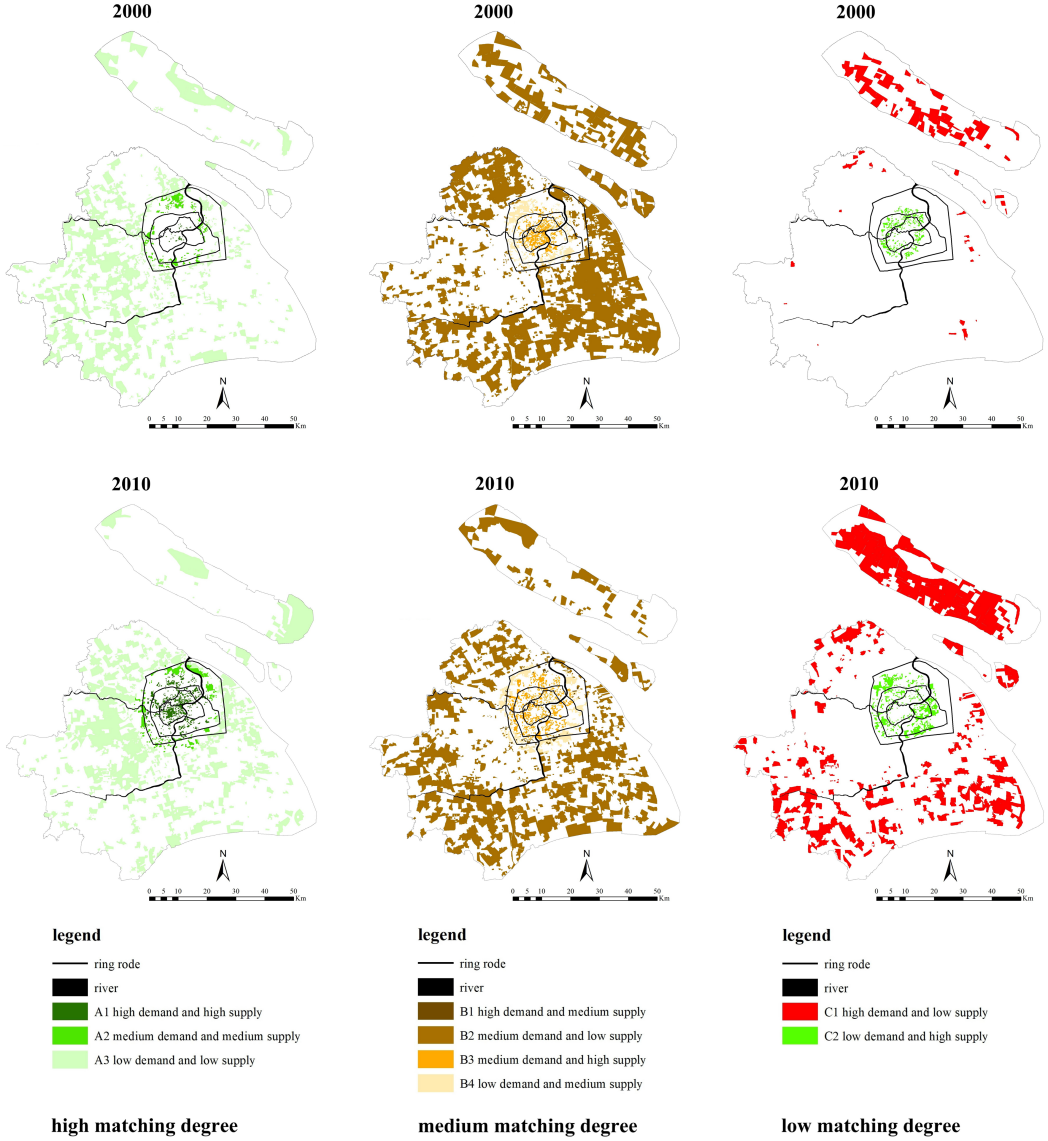
Different degrees of healthcare service supply and demand matching in 2000 and 2010.

Figure 12 shows the different matching degrees. Communities with high matching are distributed within the outer-ring. Among these communities, communities with high demand and high supply are mainly distributed within the inner-ring and from 2000 to 2010, these communities increased and expanded to within the mid-ring. Communities with medium matching are distributed in all districts of the city. Among these communities, those with higher demand levels are located in the outer suburbs, while those with lower demand levels are in the inner suburbs. The demand level of communities in the central urban area lies between that of the outer and inner suburbs. Communities with low matching are mainly distributed within the inner-ring and outside the outer-ring. Within the inner-ring, the communities had low demand and high supply, while outside the outer-ring, the communities had high demand and low supply.

### 4.3 The changing characteristics of distance of the older adult population to the closest hospital

**Figure 13:**
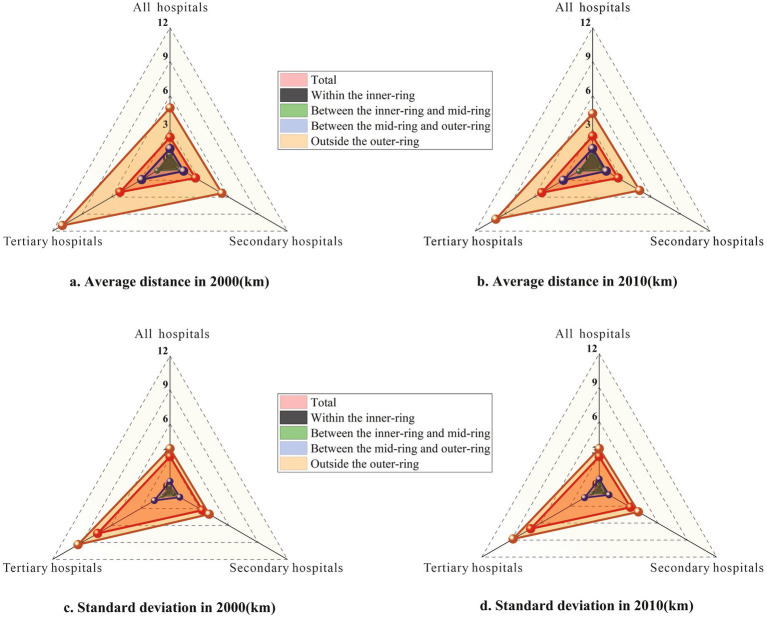
Distance (average and standard deviation) of the older adult population to the closest hospital in different zones in 2000 and 2010. **(a)** Average distance in 2000 (km). **(b)** Average distance in 2010 (km). **(c)** Standard deviation of distance in 2000 (km). **(d)** Standard deviation of distance in 2010 (km).

Although the numbers of public hospitals in the central areas and the suburban areas in Shanghai increased from 2000 to 2010, the average distance between the older adult population and the closest hospital has slightly increased due to the relocation of the older adult population to the suburban areas where sufficient healthcare services are lacking. As Figure 13 shows, the average distance was 2.3 km and 2.4 km in 2000 and 2010, respectively. The average distance to the closest tertiary hospital increased by 0.1 km and the average distance to the closest secondary hospital remained almost unchanged.

Standard deviation was used to measure the difference in distance between the older adult population and the closest hospital. As Figure 13 shows, the standard deviation of the distance between the older adult population and the closest hospital decreased. In 2010, the standard deviation of the distance between the older adult population and the closest hospital was 2.9 km, which was less than the number in 2000; and the standard deviation of the distance between the older adult population and the closest tertiary hospital was 7.0 km, which was also less than the number in 2000.

There was a great difference between distances of the older adult population in the central areas and in the suburban areas to the closest hospital. In 2010, the average distance of the older adult population who live within the inner-ring to the closest hospital was about 0.9 km, within walking distance; and the number for the older adult population who live outside the outer-ring was 4.4 km. In 2010, the average distance of the older adult population to the closest tertiary hospital to those who live outside the outer-ring was about 10 km, while the number for the older adult population who live within the inner-ring was just about 1 km. Within the inner-ring, there were more older adult population who had walking distance to the closest hospital: nearly 80% of the older adult population had walking distance to the closest hospital and more than 50% of this older adult population had walking distance to the closest tertiary hospital. However, outside the outer-ring, less than 10% of the older adult population had walking distance to the closest hospital and less than 3% of the older adult population had walking distance to the closest tertiary hospital. Moreover, in 2010, a striking 57.0% of older adults residing outside the outer-ring lived more than 10 kilometers from the closest tertiary hospital, underscoring the severe spatial inequity in access to high-level healthcare.

**Figure 14:**
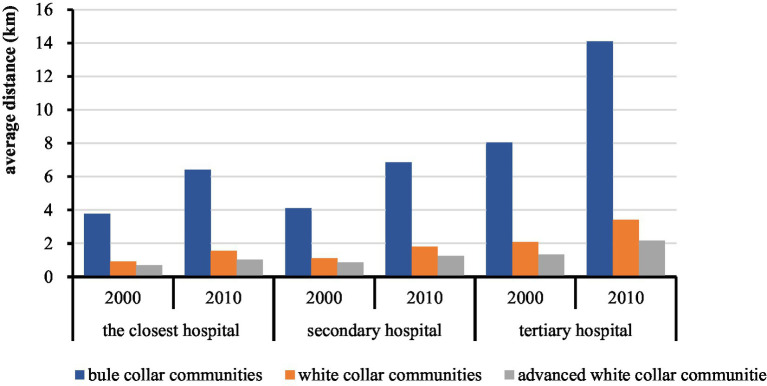
The average distance of the older adult population to the hospitals in different communities in 2000 and 2010.

There were great differences between the distances of the older adult population from different socioeconomic strata to the closest hospital. As Figure 14 shows, there are considerable differences in the distances between the older adult population in the three communities and the closest hospital; the higher socioeconomic status, the closer the average distance to the closest hospital. In 2010, the distance of the older adult population in blue-collar communities to the closest hospital was 6.4 km, while the numbers for the older adult population in white-collar and advanced white-collar communities were just about 1 km. The distance of the older adult population in blue-collar communities to the closest tertiary hospital was 14.1 km, the numbers were just about 3 km and 2 km for the older adult population in white-collar and advanced white-collar communities, respectively. Within the 10 years from 2000 to 2010, the distance of the older adult population in blue-collar communities to the closest hospital increased rapidly and to the closest tertiary hospital increased by about 6 km, indicating worse accessibility. However, there were no significant changes in the distance of the older adult population in white-collar and advanced white-collar communities to the closest hospital.

Also, within the walking distance to the closest hospital, there were fewer older adult population in blue-collar communities than in white-collar communities. In 2010, only about 1.3% of the older adult population in blue-collar communities had walking distance to the closest hospital; the numbers for the older adult population in white-collar and advanced white-collar communities were 48.5 and 56.4%, respectively. More than 80% of the older adult population in blue-collar communities had more than 5 km distance to the closest tertiary hospital. 16.6% of the older adult population in blue-collar communities had more than 10 km distance to the closest hospital, while the numbers for the older adult population in white-collar and advanced white-collar communities were less than 1%. The proportion of the older adult population in blue-collar communities who have walking distance to the closest hospital decreased from 26.4% in 2000 to 1.3% in 2010.

## 5 Discussion

Based on the comprehensive data analysis, this study provides strong evidence of significant health inequities faced by the older adult population in suburban Shanghai, despite the continuous increase in the number of healthcare hospitals and overall improvements in supply levels. First, as more and more older adult population move to the suburbs, the healthcare demand continues to rise, However, due to the concentrations of hospitals in the central areas and extensive distribution of the older adults, the healthcare services supply failed to effectively respond to the spatial shift in demand. Second, despite the increase in the number of healthcare hospitals in Shanghai, the average distance for the older adult population to the closest hospitals has shown almost no improvement. Moreover, while the redistribution of healthcare resources has improved hospital accessibility for some older adults in the suburbs, the proportion of the population within walking distance of a hospital remains relatively small. Third, there is a significant health inequity between people from different socioeconomic strata. Among the older adult population, those living in blue-collar communities have the longest average distance to the closest hospital and the lowest distribution proportion in communities with walking distance to the closest hospital. In contrast, the accessibility of healthcare for the white-collar population is considerably better, and this disparity continues to widen. This finding is consistent with global evidence that socioeconomic status (SES) is a fundamental driver of healthcare access and healthy aging (48, 49).

This study uses the neighborhood committee (*juweihui*) as the basic spatial unit. As a social area with relatively homogeneous socioeconomic status among its residents, it reflects urban residential segregation based on factors such as occupational status and household registration. The previous analysis in this study has conducted a comprehensive quantitative evaluation of equitability. Next, we will further adopt a health equity perspective to explore how China’s urban governance structure leads to the unequal distribution of healthcare resources. Specifically, we analyze how institutional mechanisms, including the healthcare system, urban development patterns, and residential segregation driven by the household registration system and housing marketization, shape the resource allocation. This approach combines “health inequality” and “health inequity,” offering a deeper understanding of the disparities in healthcare access.

The poor supply and demand match of public hospitals in the suburban areas is the result of the joint effects of governments and market mechanism. Market mechanism has been encouraged in China’s healthcare reform. For example, in the Opinion about Deepening Reform of Medical and Health Sectors issued by the central government in 2009, market force was emphasized to play a significant role in conjunction with government in establishing a national system for essential drugs and improving development policies and plans for the pharmaceutical industry (50). Public hospitals have become profit-driven and therefore insist on locating in the central areas, where have stronger consumption capacity and are more attractive to outside patients and have high profitability. Meanwhile, although operation by market and commercial principles are gradually encouraged, political forces and government interventions remain dominant in allocating healthcare resources and they have indeed made significant efforts to address health inequities. For example, Shanghai based on its “5 + 3 + 1” project opened 4 new tertiary Grade-A hospitals (the 3-A hospitals hereafter, the highest quality hospital in China) in the suburban areas (51). Shanghai has also promoted the incorporation of the suburban branch hospitals into the general hospitals to provide the suburban residents with homogeneous and high-quality medical services as in the general hospitals (52). However, given the significant role of government funding plays in operating public hospitals, locating hospitals in the suburbs could put huge pressure on the government finances due to the low profitability of the suburbs. In the sense, locating hospitals in the central areas is financially more advantageous. Further, governments at different levels may have conflicting interests over healthcare resources allocation. While the municipal government may pursue balancing healthcare resources for the whole city, the district governments in the central areas consider their own economic interests and therefore are reluctant to relocate hospitals from their jurisdiction to the suburbs.

Apart from the effects of the healthcare system, allocating more healthcare resources in the suburban areas is also challenged by China’s neoliberal urban development. In the past years, Chinese cities have built substantial high-end commodity housing in its central areas to generate more economic profit. This is often through pro-growth coalition between local governments and private developers and has resulted in fundamental reorganization of social space and demographical structure (53). For example, Shanghai has been initiating large-scale land and property-led redevelopment projects in its city core area since the 1990s. These redeveloped areas often have high-quality living standards and better and more public resources and services such as public hospitals because these elements can attract more high-income people. On the other hand, due to the high housing price in the city core areas, ordinary working class, low-income population and migrant workers, and older adult population have gradually been concentrating in the suburban areas, where have worse public resources and services (54, 55). This has intensified socioeconomic and housing inequalities in the suburban areas (56). This research demonstrates the older adult population’s worst accessibility to public hospitals especially those older adult population living in blue-collar communities in the suburban areas.

Furthermore, the segregation of the household registration system and the effects of housing marketization have jointly exacerbated the residential differentiation and spatial segregation in Chinese megacity, constituting a significant barrier to the equitable allocation of healthcare resources. Unlike the suburbanization pattern in Western countries, which is characterized by “white flight,” the suburbanization process in Shanghai has attracted a large number of residents with lower occupational status and without local household registration. Since the establishment of the socialist market economy reform objectives in 1992, household income sources have become increasingly diversified and stratified, with socioeconomic status emerging as a critical factor driving socio-spatial differentiation. Housing and labor market mechanisms have collectively contributed to a clear trend wherein residents with lower income and social prestige face increasing spatial marginalization (57). In Shanghai, analysis based on occupational indices reveals rapid white-collarization and gentrification in central-city neighborhoods. Against the backdrop of a general population dispersal toward suburbs, higher occupational groups still exhibit a strong tendency to cluster in the central city. At the same time, the household registration system further reinforces this spatial differentiation. The western suburbs of Shanghai has become a concentration area for inter-provincial migrants. These migrant settlements have expanded from north to south, progressively shifting toward the urban periphery and displaying distinct marginalization tendencies. A considerable proportion of residents in the blue-collar communities examined in this study are mostly these inter-provincial migrants without Shanghai household registration. The spatial concentration and institutional exclusion jointly exacerbate structural disadvantages in accessing high-quality public services, including healthcare resources. This mechanism of institutional exclusion compounding spatial disadvantage is not unique to China. As Guerrero and Wallace (58) demonstrated in the U. S. context, historical racism and economic inequality create a cascade of inequities for older adults of color, affecting their living arrangements, exposure to health risks, and ultimately, health outcomes. Similarly, in Shanghai, the household registration system functions as a structural determinant that predisposes a significant segment of the older adult population to healthcare marginalization.

With the growing trend of population aging in highly socioeconomically heterogeneous megacities, China needs to recognize the increasing healthcare demands of the older adult population and address the healthcare challenges they face, such as the inequitable access to public hospitals in the suburban areas. In response to these inequities, we propose several policy recommendations. For example, integrating key parameters such as “population age structure” and “proportion of vulnerable groups” into regional healthcare resource planning; establishing a dedicated fund to support the construction of suburban hospitals, providing subsidies for medical staff working in the suburb, and offering transportation assistance to patients; introducing dedicated medical bus services linking large residential communities with regional healthcare centers in underserved suburban areas; and promoting the adoption of integrated “video consultation + medication delivery” models to alleviate the travel burden of older adults. However, the perspective of health equity reveals that the fundamental causes of healthcare services injustice lie partly outside the health sector itself. The observed pattern of inequity is the outcome of central government requirements and local economic development pressures, as well as multi-groups negotiations within cities. Unless some structural features are altered, piecemeal interventions such as the increasing the number of hospitals, may benefit some residents but may not reduce inequality. As the analysis shows, the average distance to the closest hospital has merely remained stable from 2000 to 2010, while the gap between advantaged and disadvantaged groups has actually widened. Therefore, it’s also important to restructure the healthcare system to promote a people-oriented mode of governance and empower all people in decision-making and implementation processes through participatory and procedural justice, so that various stakeholders could play a role to promote recognition of the health condition of disadvantaged individuals and groups and improve policies and programs that affect equity. In conclusion, the main contribution of this study lies in going beyond the healthcare service itself to examine the structural inequity underlying apparent inequality. For rapidly expanding Chinese megacities, it is essential to reorder the urban development priorities to a certain extent to effectively address the existing imbalance in the allocation of public service resources. In addition, we appeal to continuous evaluation and research on the supply–demand dynamics of healthcare resources and their disparities across social groups in major cities, as this evidence is crucial for good urban governance.

China’s rapid urbanization has led to the emergence of megacities and expanding urban spaces. The mismatch in public service requires systematic assessment. Given the fundamental similarities in healthcare supply mechanisms across Chinese cities, this study offers valuable insights for other rapidly growing megacities. We believe that the identified imbalances and inequities should be systematically explored and evaluated. As for public service supply, merely increasing their quantity is insufficient, and more attention should be paid to the spatial distribution so as to ensure equitable access across social groups, aligning with the development goals of modern socialist international megacities. From an international comparative perspective, this research provides a practical case study of public service resource in megacity within a socialist context. It highlights both the advantages of the Chinese urban healthcare supply pattern and what needs to be improved, thereby contributing to the global comparative studies on.

This study has several limitations. First, the study only considered the hospital levels, such as secondary hospital or tertiary hospital, and ignored the quality of the healthcare services. Second, due to data limitation, the study used the straight-line distance rather than transportation distance. However, Shanghai is located in the plain of the Yangtze River Delta, which reduces the potential deviation caused by the measurement. The inequality in healthcare access would be further exacerbated when considering travel distance and mode, as disadvantaged groups have fewer transport options and face greater inconvenience due to much more reliance on public transit. The article also lacked the investigation of the subjective demand willingness of the older adult population, which represents an important direction for future research. Finally, the analysis was limited to the period of 2000–2010 due to data availability. Although Shanghai’s demographic changes have slowed since 2010, continued evaluation of both population distribution and healthcare resource allocation remains essential and it is necessary to track the changing spatial mismatches.

## 6 Conclusion

This study investigates the significant spatial inequities in public hospital accessibility for the older adult population in suburban Shanghai, linking the concepts of “health inequality” and “health inequity.” The findings highlight that the spatial distribution of healthcare resources in Shanghai has not adapted to the ongoing trends of population aging and suburbanization. As a result, a mismatch between healthcare supply and demand persists, with older adults in suburban areas facing longer distances to hospitals, especially in comparison to their counterparts in the central areas. These inequities are deeply embedded in the governance structure of China’s healthcare system and the political economic system, which prioritize resource allocation to central urban areas and higher-income groups. Consequently, simply increasing the number of hospitals is insufficient to address the root causes of these disparities. It is imperative to reexamine and restructure the institutional mechanisms of healthcare governance and urban planning to promote a more equitable, people-centered distribution of health resources. In conclusion, this study not only reveals the inequities in healthcare accessibility and their driving mechanisms, but also provides valuable theoretical foundations and policy recommendations for optimizing public services in rapidly expanding megacities.

## Data Availability

The original contributions presented in the study are included in the article/supplementary material, further inquiries can be directed to the corresponding author.

## Footnotes

1 Data refers to the registered permanent resident (*hukou*) population of Shanghai.

2 The young-old population refer to the older adult population aged 60 to 69; the middle-old population refer to the population aged 70–79; the oldest-old population refer to the population aged 80 years and above.

3 The central areas include: Jingan district, Huangpu district, Hongkou District, Changning district, Putuo district, Xuhui district, Yangpu district. The suburban areas include: Minhang district, Baoshan district, Jiading district. Outer suburbs include: Jinshan district, Songjiang district, Qingpu district, Fengxian district, Chongming district.

4 The indicator used by service supply intensity of each hospital to a *juweihui* is the distance from the *juweihui* to the hospital; *juweihui* which is closed to hospitals is considered to receive more intensive healthcare service supply.

5 The distance between a *juweihui* to all hospitals is summed up to become the cumulative service supply intensity of all hospitals in Shanghai received by the *juweihui*; the smaller the summed distance, the greater the cumulative service supply intensity of hospitals, and the higher indicators. The cumulative service supply intensity of all the hospitals in the city received by the *juweihui* includes the secondary and tertiary hospitals. Considering the obvious difference in the quality of healthcare services between secondary and tertiary hospitals, the cumulative service supply intensity is the weighted sum of the supply intensity of the two types of hospitals.

6 As data on the health status of the older adult population were lacking in 2000, a weighted average of the remaining three indicators were actually used. Because the health status index is consistent with the aging and aging index, the absence of the health status of the older adult population in 2000 has little impact on the results.

7 The method of assigning points to occupational categories is as follows: managers of all kinds (7 points), professionals (6 points), clerical personnel (5 points), commercial service personnel (4 points), production, transportation and equipment operation related personnel (3 points), agriculture, forestry, animal husbandry and fishery related personnel (2 points) and unemployed people (1 points). Then, according to the occupation index in different communities, the study categorizes communities in Shanghai as blue-collar communities (index lower than 3.5), white collar communities (index between 3.5 and 4.8) and advanced white-collar communities (index higher than 4.8).

8 A negative index means that the supply is relatively greater than the demand and healthcare services is in good conditions. A positive index means the demand is relatively greater than the supply and healthcare services is not in good conditions. If the index is zero, it means a relatively balanced relationship between the supply and demand.

9 We used the neighborhood analysis tool in Geographical Information System to calculate the distance, that is, the distance between the geometric center of the communities and the closest secondary and tertiary hospitals. Then we used EXCEL to calculate the weighted average distance and weighted standard deviation.
